# Dimer/tetramer motifs determine amphiphilic hydrazine fibril structures on graphite

**DOI:** 10.3762/bjnano.3.75

**Published:** 2012-09-19

**Authors:** Loji K Thomas, Nadine Diek, Uwe Beginn, Michael Reichling

**Affiliations:** 1 Fachbereich Physik, Universität Osnabrück, Barbarastr. 7, 49076 Osnabrück, Germany; 2 Institut für Chemie, Universität Osnabrück, Barbarastr. 7, 49076 Osnabrück, Germany

**Keywords:** fibrils, graphite, hydrazide, hydrazine, interface, self-assembly, STM

## Abstract

Fibril structures are produced at a solvent–graphite interface by self-assembly of custom-designed symmetric and asymmetric amphiphilic benzamide derivatives bearing C_10_ aliphatic chains. Scanning tunnelling microscopy (STM) studies reveal geometry-dependent internal structures for the elementary fibrils of the two molecules that are distinctly different from known mesophase bulk structures. The structures are described by building-block models based on hydrogen-bonded dimer and tetramer precursors of hydrazines. The closure and growth in length of building units into fibrils takes place through van der Waals forces acting between the dangling alkyl chains. The nanoscale morphology is a consequence of the basic molecular geometry, where it follows that a closure to form a fibril is not always likely for the doubly substituted hydrazine. Therefore, we also observe crystallite formation.

## Introduction

One-dimensional micro- and nanostructures of organic compounds are important for solution-processable organic electronic devices [1–3], and electron transport through organic molecules is also the basis for a large number of biological processes [4]. Organogelators have a tendency to form nanofibril structures in the bulk phase and, therefore, recently aroused much interest in the context of nanoelectronics [5]. Except for biological systems, organogel structures are the only synthetic self-organized linear entities, facilitating the construction of functional arrangements up to millimetre dimensions [6]. With suitable functional moieties, they can guide ions, electrons or even photons and can serve as interconnects when integrated into electronic or bioelectronic devices [1,5,7]. Further progress in this area is mostly limited by low charge-carrier mobility and the mostly amorphous local packing. Therefore, it is essential to synthesize optimized materials, explore supramolecular routes towards new functional structures, and understand processes of structure formation at interfaces [8–9].

The knowledge about the internal structure of the columns (fibres) in bulk columnar mesophases depends mostly on X-ray techniques, which suffice for many purposes [7,10–11]. However, information in real space, as provided by scanning tunnelling microscopy (STM), offers unparalleled advantages to the synthesis chemist who strives to functionalize fibrils that are one-dimensional structures with only a few nanometres in diameter. The control of supramolecular self-assembly to achieve functional nanostructures depends on careful design at the molecular level, and elucidation of their internal structure is important in aiding the design and to increase the sophistication of the building units.

**Figure 1:**
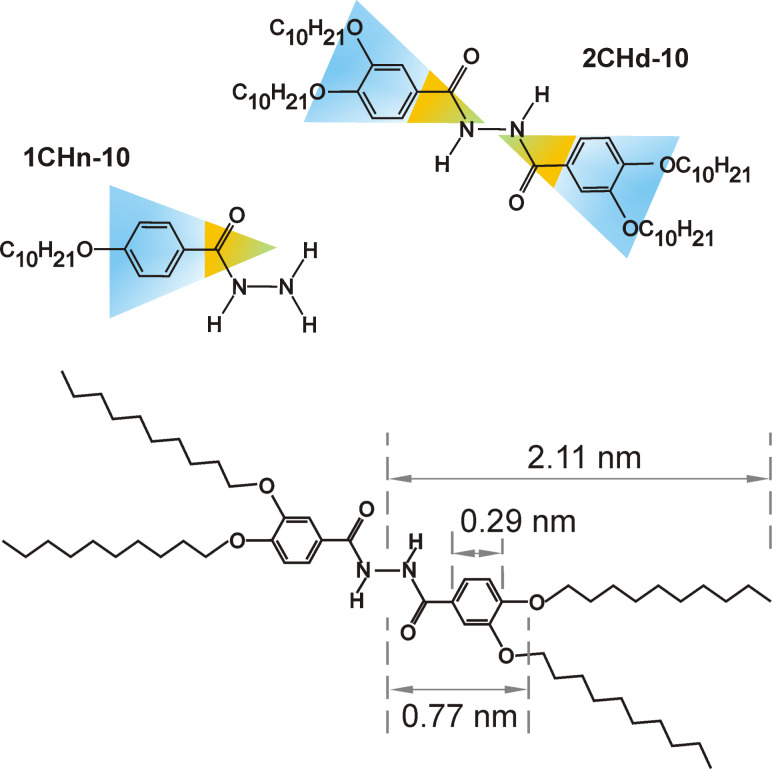
Structure models of **2CHd-10** (inversion symmetry) and **1CHn-10** (asymmetric). The coloured region represents the "wedge"-shaped nature of the molecules. The molecular dimensions given for **2CHd-10** are derived from [14–15].

Many low-molecular-weight, wedge-shaped amphiphilic molecules are known to form columnar mesophases [3,6]. X-ray diffraction and scattering techniques have widely been used to decipher their internal molecular arrangements, which generally suggest a stacking of mesogenic “discs” leading to column formation [7,12]. We investigate the self-assembled fibril structures of two custom-designed amphiphilic gelator molecules: *N*,*N*′-bis[3,4-bis(decyloxy)benzoyl]hydrazine (**2CHd-10**) and [4-(decyloxy)benzoyl]hydrazine (**1CHn-10**) on the graphite (0001) surface (Figure 1 and Experimental section). As the alkyl chain length is known to influence column formation in the bulk, the length of alkyl chains for both molecules is kept identical such that the focus of the study is solely on the geometry/symmetry aspect [6]. In Figure 1, a wedge shape is shown superimposed on the molecular structure, where the amide moieties are at the tip of the wedge and the alkoxy chains at the tail. In general, the molecular geometry of the mesogens is decisive for the generation of columnar mesophases in the bulk, i.e., the mesogens should be wedge-shaped. The wedges can form a disc with their tips all directed to the centre; for example, six such wedge pieces may lead to a hexagonal columnar mesophase. Further stacking of discs in a face-to-face configuration leads to columns [6,12–13].

To evaluate the possibilities of self-assembly of **2CHd-10** and **1CHn-10** molecules on HOPG, we note that their amide functionalities can efficiently stabilize structures through intermolecular hydrogen bonds. On the other hand, the alkyl chains are expected to promote self-assembly into extended structures through interchain van der Waals interactions as well as adsorption on HOPG due to their epitaxial match with the C–C bonds of graphite [16]. The structures produced, may, however, generally depend on a complex interplay of many weak interactions. The molecules are prototypes for symmetric and asymmetric hydrazine species, where **2CHd-10** represents two 2CHn-10 molecules linked together such that the amide functionality is not a head group but a central part, which is expected and found to have considerable influence on the self-assembly behaviour. We observe that the molecules self-assemble into one-dimensional structures at the solution/HOPG interface, which are distinctly different from those of the “disc-stacking” pattern in the bulk. The structures of elementary fibrils are explained by dimer or tetramer precursors followed by fibril formation through van der Waals interactions between alkyl chains. The molecular geometry plays a crucial role in deciding the type of oligomer precursor: self-assembly is based on dimer building blocks for **2CHd-10**, but tetramer building blocks for **1CHn-10**. It appears that the large-scale morphologies result as a direct consequence of the type of oligomer precursors they form.

As a substrate, highly oriented pyrolytic graphite (HOPG) is favoured in STM studies due to its high electrical conductivity, atomic flatness, chemical inertness and here also for its hydrophobic nature. Hydrophilic substrates could hinder the self-assembling ability of the molecules by strongly interacting with their amide functionalities and forcing them to lay flat on the surface. To study adsorbate architecture on an electrically conducting substrate in real space, scanning tunnelling microscopy (STM) is the experimental technique of choice. Although STM has been highly successful in atomic/submolecular probing of planar structures [17–18] and their dynamics [19], when it comes to one-dimensional structures, it has not shown the same level of efficacy [20]. Particularly for high-resolution imaging in an ambient/solution environment, data acquisition becomes an exhausting and time-consuming process. Difficulties arise from the requirement of having single-digit nanometre-wide isolated strands and locating them on a millimetre area substrate; thermal drift; movement/perturbation induced by tip motion and tip contamination [21]; the nonplanar nature of components within individual fibril units; and the presence of dangling alkyl chains. High-resolution STM imaging of 1-D structures has been successful in studying films of strands [22–24] and innate graphitic structures [25–27], but less so with isolated organic strands. Some reports of STM imaging to obtain high-quality images of strands include those of polypropylene [28], molecular chains of magnetic molecules [29], silicon nanowires [30], and DNA/biomolecules [31–32].

With regard to STM imaging of 1-D structures on HOPG, one should be wary of innate graphitic artefacts and 1-D fibre-like structures present on bare HOPG surface, mostly occurring as a result of cleaving [25–27]. Although, graphitic artefacts may show strikingly close resemblance to molecular fibrils, the two species can be distinguished from each other. Care has been practised at all stages during STM imaging as well as analysis to establish the adsorbate origin of the reported structures clearly. The ambiguity can be excluded due to the capability of **1CHn-10** and **2CHd-10** to produce fibrils (as evident from the AFM images), the absence of grain boundaries and single/multiple steps near the molecular wires [25] (which are the two most important causes for their appearance), and the discrepancy in periodicities between molecular structures and reported innate graphitic fibril-like objects [25–27]. Further, we note that all reported high-resolution graphitic strands exhibit a replica-type arrangement with strands appearing as a replica of each other with bright blobs aligned perfectly on a line against the long axis, while for our molecular structures such a replica pattern is not observed. We have extensively studied innate planar and fibril-like graphitic artefacts at large scales (micrometres) as well as with high-resolution (few nanometres) and found the graphitic structures to be similar to those reported previously but different from the fibril structures reported here.

## Experimental

### STM/AFM imaging

For sample preparation, solutions of different concentrations for each of the molecules were prepared by dissolving the respective sample in 1,2,4-trichlorobenzene (C_6_H_3_Cl_3_, dielectric constant 2.2, boiling point 214 °C, 99% pure, Sigma-Aldrich Laborchemikalien GmbH, Seelze, Germany) in a dilution series in steps of 1/10. Higher concentrations often exhibit a gel-like character. The solution was usually sonicated or oven-heated to 45–50 °C for five to fifteen minutes before being applied to a freshly cleaved sample of highly oriented pyrolytic graphite (HOPG, ZYB grade, SPI supplies, West Chester, PA, USA). First, a suitably dilute concentration for STM imaging was found that leaves fibrils on HOPG without much clustering or bundling. Then, the particular concentration was repeatedly used for obtaining high-resolution STM images.

STM images were taken in the constant current mode under ambient conditions with a compact STM (Easyscan, Nanosurf AG, Liestal, Switzerland). Mechanically sharpened Pt/Ir (80/20) wires (Goodfellow Cambridge limited, Huntingdon, United Kingdom) were used as tips. Prior to measurements on molecular layers, the bare HOPG substrate was imaged to ensure the quality of the STM tip and the cleanliness of the substrate surface. By imaging the atomic structure of the bare graphite, the scanner was calibrated at regular time intervals so that the precision of measurements was solely limited by thermal drift. The entire scan area was also imaged before molecules were deposited, to check for graphite artefacts. The ambient temperature was stabilized to be within ±1.0 °C of room temperature, and the scanner was always given time to thermally equilibrate and mechanically relax, to reduce thermal drift and piezo creep to a minimum during measurements. Furthermore, images used for structural analysis were those with minimal thermal drift, and a drift correction was done whenever feasible.

For imaging of molecular structures, the tip was retracted slightly, and a drop of the solution was applied onto the basal plane of HOPG to form a meniscus between the tip and the surface. Imaging was performed at the solution–solid interface where typical operating conditions were *V*_t_ = 1.3 V tunnelling voltage and *I*_t_ = 0.60 nA tunnelling current for the molecule and 0.05 V at 1.00 nA for imaging the bare graphite substrate. Occasionally, another preparation method was used, i.e., a drop of the solution was deposited on HOPG and imaging was started after complete evaporation of the solvent. Similar results were obtained by employing either preparation method, and once formed, the structures remained stable for many hours to days.

Images represent raw data unless otherwise stated, and flattening was done only for large area images, by using the WSxM software [33]. A compact AFM (Easyscan, Nanosurf AG, Liestal, Switzerland) in contact-mode was used to characterize the nanoscale morphology. Silicon cantilevers (Nanosensors) with force constants in the range from 0.2 to 0.4 N/m were employed, and the images were taken under ambient conditions at a scanning rate of 1–3 lines/second with a typical force setpoint of 25 nN. Topographic data were recorded simultaneously in trace and retrace to check for scan artefacts. From clear solutions, imaging was done after a complete evaporation of the solvent. For concentrated solutions, solvent remained partially trapped within the gel network of fibrils during imaging.

### Chemical syntheses

#### Materials and techniques

Methyl 4-hydroxybenzoate (99%, Sigma Aldrich), methyl 3,4-dihydroxybenzoate (97% Alfa Aesar) 1-bromodecane (98%, Alfa Aesar), potassium iodide (99.5%, Fluka), potassium carbonate (99%, Sigma Aldrich), potassium hydroxide (85–100%, Sigma Aldrich), hydrochloric acid (37%, Sigma Aldrich), thionylchloride (98%, Sigma Aldrich), hydrazine monohydrate (98%, Alfa Aesar) were used for the chemical syntheses. ^1^H NMR (500 MHz) and ^13^C NMR (125 MHz) were measured on a Bruker Avance DPX-250 spectrometer, tetramethylsilane (TMS) was applied as an internal standard in deuterated chloroform at 20 °C. Melting points were measured on a Netzsch DSC 204 Phoenix differential scanning calorimeter (DSC). About 10 mg of sample was used. In all cases, the heating and cooling rates were 10 °C/min. Indium and cyclohexane were used as calibration standards. IR spectra were measured on a Bruker Vertex 70 FT infrared spectrometer, equipped with a MVP Star ATR reflection device.

#### [4-(Decyloxy)benzoyl]hydrazine (**1CHn-10**)

**Synthesis of methyl 4-(decyloxy)benzoate (1):** Methyl 4-hydroxybenzoate (15.215 g; 100 mmol) was dissolved in 500 mL cyclohexanone, and 24.3 g (110 mmol) 1-bromodecane, 41.3 g (30 mmol) potassium carbonate, and 0.5 g potassium iodide were added and heated under reflux for 5 h under a nitrogen atmosphere. The reaction mixture was filtered hot and concentrated on a rotary evaporator. After recrystallization from 600 mL MeOH/EtOH (2/1), a white wax-like solid was obtained. Yield: 24.3 g (83%); mp 45 °C (lit.: 44–45 °C); ^1^H NMR (CDCl_3_) δ 0.916 (t, 3H, -CH_3_), 1.337 (m, 12H, -CH_2_-), 1.458 (m, 2H, -C**H_2_**-CH_2_-CH_2_-O-), 1.812 (m, 2H, -C**H_2_**-CH_2_-O-), 3.896 (s, 3H,-COO-CH**_3_**), 4.019 (t, 3H, -CH_2_-O-), 6.91 (d, 2H, aromatic), 8.0 (d, 2H, aromatic).

**Synthesis of [4-(decyloxy)benzoyl]hydrazine (1CHn-10)***:* Compound **1** (10 g; 34 mmol) was dissolved in 20 g pentanol, and 20 g hydrazine monohydrate was added and heated under reflux at 180 °C for 6 h; the mixture was poured into 200 mL cold MeOH and filtered. The precipitate was washed two times with 50 mL cold MeOH. Afterwards recrystallization in MeOH, 5.3 g (53%) of a white solid was obtained. IR ν: 3319, 3221, 3168, 3066, 3022, 2957, 2920, 2872, 2855, 1645, 1618, 1575, 1506, 1477, 1394, 1352, 1304, 1253, 1188, 1172, 1115, 1030, 987, 835, 652 cm^−1^; ^1^H NMR (CDCl_3_) δ 0.88 (t, 3H, -CH_3_), 1.3 (m, 12H, -CH_2_-), 1.4 (m, 2H, -C**H_2_**-CH_2_-CH_2_-O-), 1.75 (m, 6H, -C**H_2_**-CH_2_-O-), 2.25 (broad, 3H, -NH-NH_2_), 3.98 (t, 2H, -CH_2_-O-), 6.88 (d, 2H, aromatic), 7.75 (d, 2H, aromatic). **1CHn-10** has been mentioned as a synthetic intermediate in several reports [34–36].

#### *N*-*N′*-bis[3,4-bis(decyloxy)benzoyl]hydrazine (**2CHd-10**)

**Synthesis of methyl 3,4-bis(decyloxy)benzoate (3):** Methyl 3,4-dihydroxybenzoate (6.0 g; 32.94 mmol) was dissolved in 200 mL cyclohexanone, and 16.0284 g (72.468 mmol) 1-bromooctane, 13.658 g (98.82 mmol) potassium carbonate and 0.2 g potassium iodide were added and heated under reflux for 5 h under a nitrogen atmosphere. The reaction mixture was filtered hot and concentrated on a rotary evaporator. After recrystallization from 200 mL MeOH/EtOH (2/1), a white wax-like solid was obtained. Yield: 11.3164 g (76.6%); *R*_f_ 0.56 (CH_2_Cl_2_); ^1^H NMR (CDCl_3_) δ 0.885 (t, 6H, -CH_3_), 1.28 (m, 24H, -CH_2_-), 1.48 (m, 4H, -CH_2_-CH_2_-CH_2_-O-), 1.83 (m, 4H, -CH_2_-CH_2_-O), 3.88 (s, 3H,-COO-CH**_3_**), 4.04 (m, 4H, -CH_2_-O-), 6.885 (d, 1H, aromatic.), 7.593 (d, 1H, aromatic), 7.71 (d,1H, aromatic).

**Synthesis of 3,4-bis(decyloxy)benzoic acid (4):** Compound **3** (8.0014 g; 17.8 mmol) was dissolved in 350 mL boiling EtOH, and a solution of 11.2 g (200 mmol) KOH in 25 mL water was added and heated under reflux for 4 h. The reaction mixture was poured into 1 L distilled water, acidified with hydrochloric acid to pH 1, and stirred for 1 h. Afterwards the precipitate was filtered and recrystallized from acetone. White crystals (7.6346 g; 98.7%) were obtained. ^1^H NMR (CDCl_3_) δ 0.885 (t, 6H, -CH_3_), 1.319 (m, 24H, -CH_2_-), 1.481 (m, 4H, -C**H_2_**-CH_2_-CH_2_-O-), 1.84 (m, 4H, -C**H_2_**-CH_2_-O-), 4.051 (m, 4H, -CH_2_-O-), 6.89 (d, 1H, aromatic.), 7.5935 (d, 1H, aromatic), 7.72 (d, 1H, aromatic).

**Synthesis of 3,4-bis(decyloxy)benzoyl chloride (5):** Compound **4** (5.0215 g; 11.55 mmol) was heated under reflux with 25 mL thionylchloride and 3 mL DMF for 2 h. The solvent was evaporated in vacuum at 60 °C. Finally 2.279 g (43.36%) of white crystals were obtained after recrystallization three times in dry acetone. ^1^H NMR (CDCl_3_) δ 0.878 (t, 6H, -CH_3_), 1.282 (m, 24H, -CH_2_-), 1.463 (m, 4H, -C**H_2_**-CH_2_-CH_2_-O-), 1.833 (m, 4H, -C**H_2_**-CH_2_-O-), 4.045 (m, 4H, -CH_2_-O-), 6.85 (d, 1H, aromatic.), 7.582 (d, 1H, aromatic), 7.745 (d,1H, aromatic).

**Synthesis of *N*-*N′*-bis[3,4-bis(decyloxy)benzoyl]hydrazine (2CHd-10)**: Compound **5** (2.2644 g; 5 mmol) was dissolved in 50 mL dry dioxane, 20 mL dry THF and 2 mL benzene, then 0.25 g (5 mmol) hydrazine monohydrate was added and stirred for 24 h. The precipitate was dissolved in 400 mL CHCl_3_ and washed two times with 400 mL concentrated NaCO_3_ in H_2_O and also with H_2_O. The organic phase was concentrated on a rotary evaporator and freeze dried from benzene. Yield: 1.9715 g (91.12%) white solid; ^1^H NMR (CDCl_3_) δ 0.893 (m, 12H, -CH_3_), 1.318 (m, 48H,-CH_2_-), 1.438 (m, 8H, -C**H_2_**-CH_2_-CH_2_-O-), 1.82 (m, 8H, -C**H_2_**-CH_2_-O-), 4.011 (m, 8H, -CH_2_-O-), 6.85 (d, 2H, aromatic), 7.437 (d, 4H, aromatic.), 9.464 (broad, 2H, -NH). IR ν: 3160, 2957, 2920, 2849, 1600, 1566, 1516, 1472, 1458, 1394, 1267, 1220, 1143, 1119, 1070, 1020, 990, 866, 746, 721, 63 cm^−1^.

## Results and Discussion

**Figure 2:**
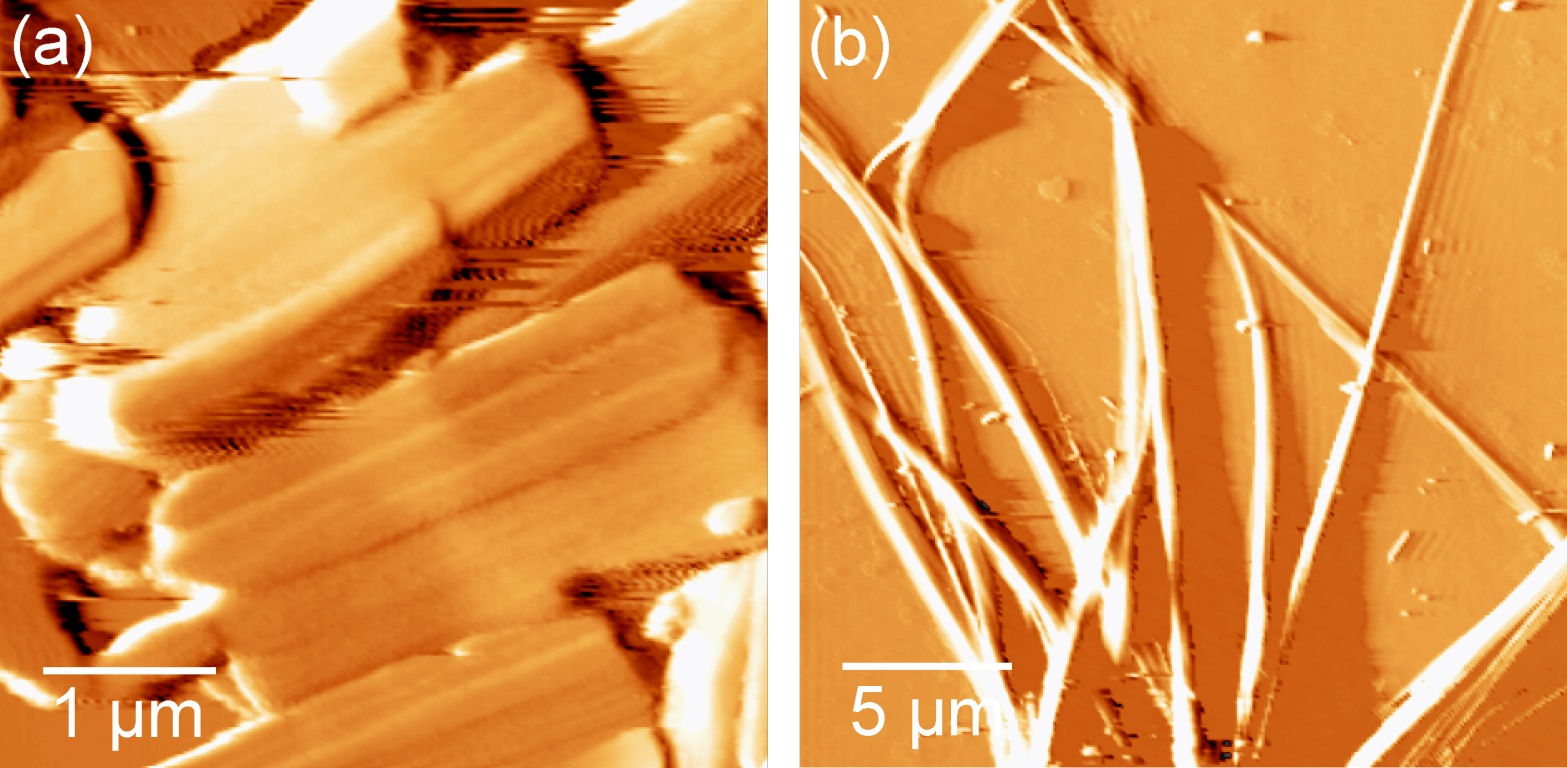
AFM images of randomly oriented (a) **2CHd-10** crystallites and (b) **1CHn-10** fibril bundles on HOPG obtained in high-concentration solutions.

**2CHd-10** is expected to facilitate column formation in the bulk due to its partial disc-like design (Figure 1) already bestowed upon synthesis. It is expected to self-assemble into a complete disc and thereupon to a stacked arrangement of discs. It has been reported that symmetrically substituted methyloxy-3CHd-1 and ethyloxy-3CHd-2 form crystalline compounds that melt above 177 °C, while 3CHd with longer chains as well as **2CHd-10** form a columnar, hexagonal, disordered (*C_hd_*) mesophase in the bulk [6]. AFM images in Figure 2 show the morphology of **2CHd-10** and **1CHn-10** on HOPG after deposition from high-concentration solutions: around 2.4 wt % for **2CHd-10** and 2.8 wt % for **1CHn-10**. Albeit **2CHd-10** possessing a particularly favourable geometry for column formation in the bulk, its morphology on the graphite surface is that of fibrillar crystallites of varied lengths rather than pure fibrils, as shown in Figure 2a.

**Figure 3:**
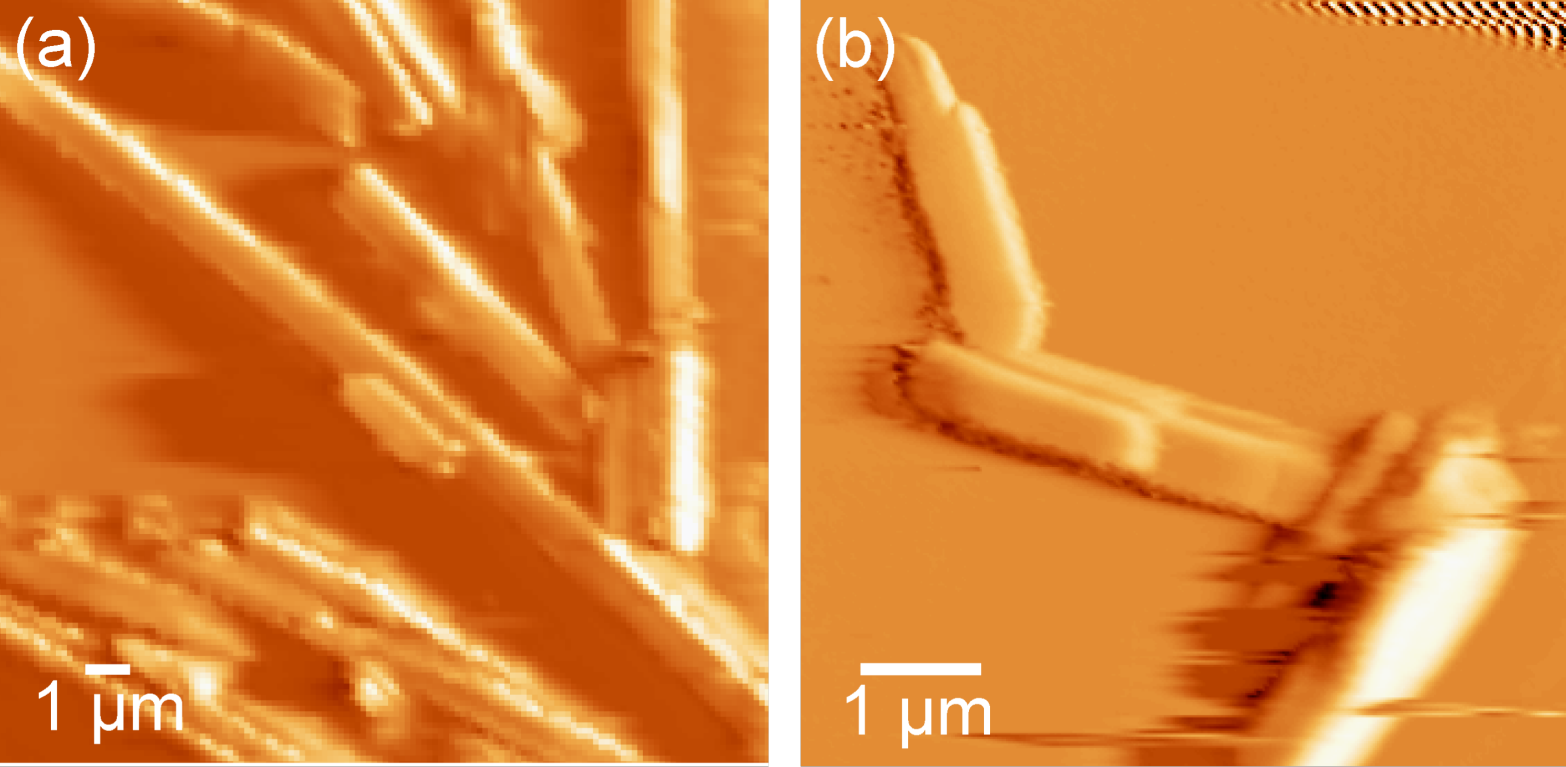
AFM images of (a) **2CHd-14** and (b) **2CHd-6** on HOPG taken to demonstrate the capability of the **2CHd-n** series to produce fibrillar crystallites.

Intrigued by the crystallite morphology of **2CHd-10**, we investigated its chain length variants, namely **2CHd-6** and **2CHd-14**, by AFM imaging, which vindicates their fibrillar-crystallite nature, with **2CHd-14** seeming to produce the longest fibres amongst the three, as demonstrated in Figure 3. On the other hand, **1CHn-10** deviates from the wedge-shape due to having only one alkoxy chain, but optical microscopy and AFM images reveal that **1CHn-10** is capable of forming fibril assemblies extending up to several tens of micrometres, as evident from Figure 2b. Note that **1CHn-10**, however, offers an additional hydrogen-bonding site (believed to enhance column ordering in the bulk). An explanation for this seemingly contrasting behaviour of **1CHn-10** and **2CHd-10** critically depends on the knowledge of the respective elementary fibril structures.

**Figure 4:**
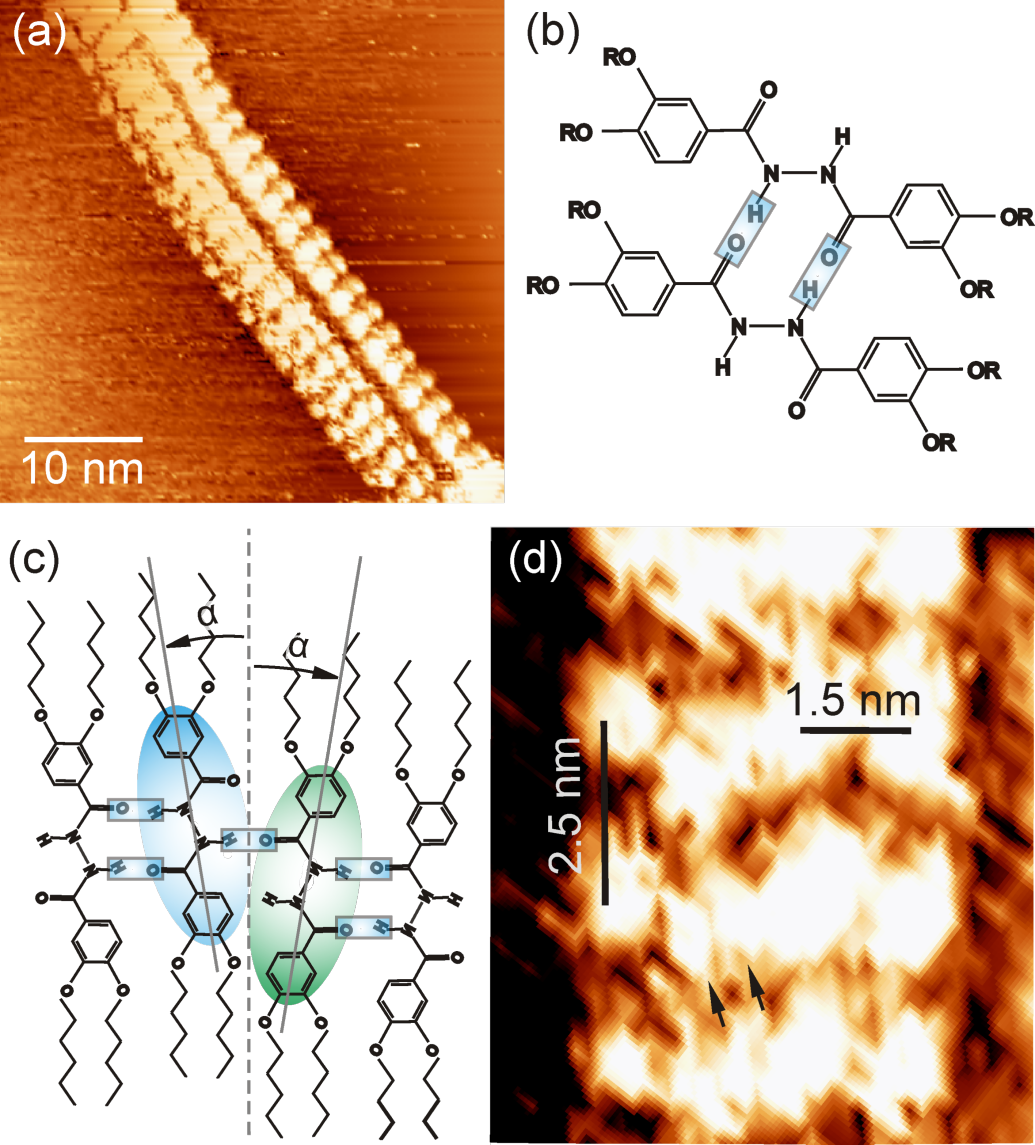
(a) STM image of two adjacent **2CHd-10** elementary fibrils on HOPG. Imaging parameters are *V*_t_ = 1.3 V and *I*_t_ = 0.6 nA. (b) Structure of a **2CHd-10** dimer. (c) Dimer arrangement in fibril. (d) Magnified view of the left fibril from (a) revealing details of its internal structure.

To investigate the structure formation of **2CHd-10** on HOPG at a molecular scale, a drop of a dilute solution of **2CHd-10** (≈0.24 wt %) dissolved in 1,2,4-trichlorobenzene (C_6_H_3_Cl_3_) was deposited on HOPG and the liquid/solid interface searched for the thinnest fibrils by STM. The use of dilute solutions for STM studies is prompted by the requisite of locating isolated elementary strands on the substrate. Fibre bundles are oriented mostly randomly but isolated elementary fibrils follow low-index graphite surface directions. Generally, the length of isolated fibrils exceeds the scan range of the STM (≈800 nm). Figure 4a is a typical STM image showing a bundle consisting of two elementary fibrils, while Figure 4d is a close-up of the left fibril taken to reveal the internal structure and its dimensions.

Having a width of 5.6 nm, the fibril consists of bright blobs arranged side-by-side in a zigzag pattern that slightly varies along the fibril. Bright blobs can sometimes be resolved into two elliptical features that are about 1.4 nm long (arrows in Figure 4d). Assuming that electron-rich delocalized π clouds of the aromatic rings dominate the image contrast [37], the bright blobs are interpreted as hydrogen-bonded dimers, as shown in Figure 4b. Note that the distance between adjacent bright blobs is 1.5 and 2.5 nm in directions perpendicular and parallel to the fibril axis, which is much larger than the interstack distance of 0.35 nm observed in mesophase columns in the bulk [10]. This means that the fibrils cannot be explained by a stacked structure stabilized by π* orbital overlap of adjacent aromatic rings. The measured heights (brightness) of individual bright blobs in a zigzag vary slightly, which could be a convolution of electronic and topographic effects implying the three-dimensional nature of the structure hidden in the topographic image.

**Figure 5:**
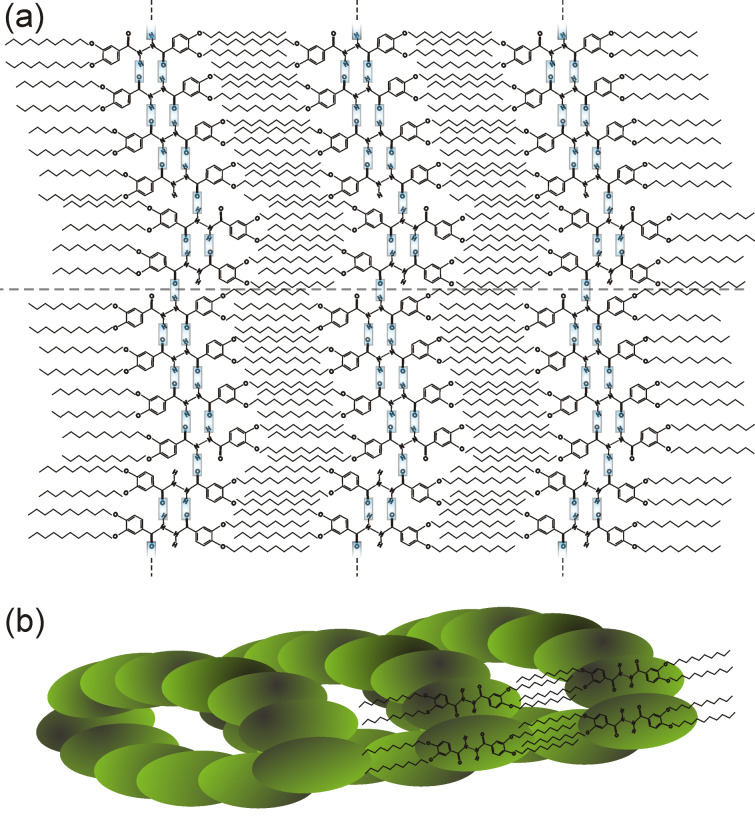
(a) Planar-sheet model (net) of a **2CHd-10** fibril section. The dashed line is drawn parallel to the fibrillar axis. (b) 3-D representation of a fibrillar fragment.

A structural model is proposed for the **2CHd-10** fibril based on dimer precursors involving mainly hydrogen bonding along the circumference and van der Waals bonding between interdigitated dangling alkyl chains along the fibril axis, as shown in Figure 5a as a “net” of the fibril. Note that the periodicity of the structure along the fibril axis, predicted by this model and the molecular dimensions given in Figure 1, is in reasonable agreement with the periodicity of 2.5 nm observed in experiments (see Figure 4d). Figure 5b is a 3-D model in which individual ellipsoids represent **2CHd-10** molecules. The ellipse representing the bright STM contrast feature defines the symmetry axis of the molecule while the fibril axis is defined by the direction of the alkyl chains, as illustrated in Figure 4c. The tilt of α = 9° between the molecular axis and the fibril axis is determined by aligning the alkyl chains while reducing their bending with respect to the aromatic rings to a minimum.

One could construct a perfectly planar molecular layer of surface-filling molecules based on the described construction principles. However, zigzag structures result from defects introduced by dimers flipped by 180° around the fibril axis (shaded blue and green in Figure 4c). As evident from Figure 4c and Figure 5a, such a flipped molecule can form only one hydrogen bond with the neighbouring dimer and is tilted in the opposite direction yielding a step in the molecular contour of the hydrogen-bonded units. The loss of one hydrogen bond at defect sites is partially compensated by additional interdimer hydrogen bonds (see Figure 5a). Due to a perfect interdigitation of alkyl chains, the fibril has only a weak interaction with the substrate. We speculate that defects introduce internal stress resulting in a small bending of the initially planar sheet. A fibril fragment may result if the specific zigzag structure facilitates a hydrogen-bond closure from open hydrogen bonds, as indicated (unbonded H atoms at the top of the net and O atoms at the bottom) in Figure 5a.

Fibril fragments can grow with different diameters depending on the number of molecules in the sheet, while the detailed zigzag structure determines whether a closure is possible or not. Once a closed fragment is formed, the fibril can easily grow along its axis by the attachment of more dimer units. Planar fragments that are unable to close may still grow axially leading to fibrillar crystallites, as the axial growth mechanism is basically the same as that for a closed net, i.e., through van der Waals interactions. It can also be conjectured that a closure is most plausible for nets with a small diameter, whereas large nets may lie flat on the surface and grow as crystallites. It is worth noting that the model described here displays striking similarities with the bulk mesophase fibrils in its basic constitution. First, from X-ray data for the bulk mesophase fibrils, the number of molecules per column (a disc) cross section is also found to be two, i.e., a dimer [6]. Second, the periphery of the fibril cross section in the columnar hexagonal disordered mesophase consists of six dimer units just as for the six-membered dimer fibril cross section shown in Figure 5b.

**Figure 6:**
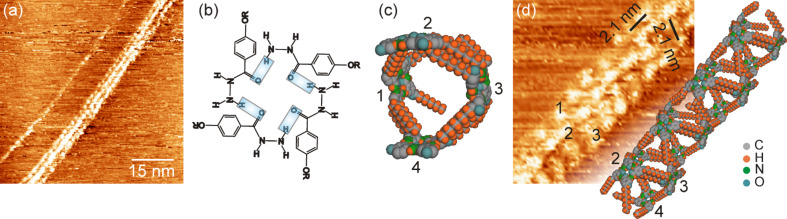
(a) STM image showing a single-strand and a three-strand fibril of **1CHn-10** on HOPG. Imaging parameters are *V*_t_ = 1.29 V and *I*_t_ = 0.69 nA. (b) Structure of a **1CHn-10** tetramer. (c) Model of the repeating unit of a three-strand fibril formed by four tetramers. (d) Magnified view of the three-strand fibril section from (a) and the corresponding space-filling model based on the construction principle discussed in the text.

Next, we investigated the structure formation of **1CHn-10** on HOPG at the molecular scale. Figure 6a shows an STM image taken after a drop of a dilute solution of **1CHn-10** in 1,2,4-trichlorobenzene (≈0.29 wt %) had been deposited on HOPG. Again fibrils with a length covering the entire STM scan range are observed. Unlike for **2CHd-10**, we observe single-strand and three-strand fibrils (top view) as shown in Figure 6a. As revealed by Figure 6d, the periodicity of bright blobs along the fibril bundle as well as the lateral distance between strands is 2.1 nm. The individual bright blobs of about 1.5 nm appear as rather elusive features in single-strand fibrils. We assume hydrogen-bonded tetramers, as shown in Figure 6b, to be the building blocks for the strand structure. The tiny single-strand fibrils yielding only unstable STM contrast may simply be linear arrangements of tetramers between overlapping alkyl chains.

The three-strand geometry appears as a much more rigid structure, and a plausible repeating unit for it is illustrated in Figure 6c in which four tetramer building blocks form a ring stabilized by van der Waals interactions between dangling alkyl chains. This ring is a highly symmetric unit in which the tetramer aryl cores (assuming the tetramers to be planar) appear pairwise parallel (tetramers 1║3 and 2║4), with 2 and 4 displaced from 1 and 3 by half the periodicity along the fibril axis. In a projection perpendicular to tetramer 2, tetramer 4 appears precisely below tetramer 2, and 1 and 3 appear symmetrically at the sides of 2 where the connecting lines 1–2 and 2–3 enclose an angle of 120°. In such a ring unit, 8 of the 16 available alkyl chains are van der Waals bonded to each other while four are dangling at one side of the ring and four at the other side (two alkyl chains each from 2 and 4 are not shown in Figure 6c).

Unlike **2CHd-10**, here no condition is to be met for the closure of **1CHn-10** tetramers to form a ring. Due to the symmetry of the ring unit, the dangling alkyl chains are at the right positions to connect to alkyl chains of a following ring in the very same manner the tetramers in a ring unit are bonded internally, i.e., through van der Waals interactions. This interaction between subsequent tetramer rings leads to their growth into a linear chain. Hence, a string of ring units yields a fibril with a well-defined diameter and a saturation of all possible van der Waals bonds between alkyl chains. Assuming the strand of tetramers numbered 4 is lying flat on the HOPG surface, the fibril structure appears in the above-mentioned projection, and tetramers 1, 2 and 3 appear as bright blobs in the STM image of Figure 6d ordered in a linear herringbone arrangement exhibiting the 120° angle. A 3-D space-filling model for the fibril is shown in Figure 6d, which is a periodic structure of the ring unit of Figure 6c at a periodicity of 2.1 nm.

**Figure 7:**
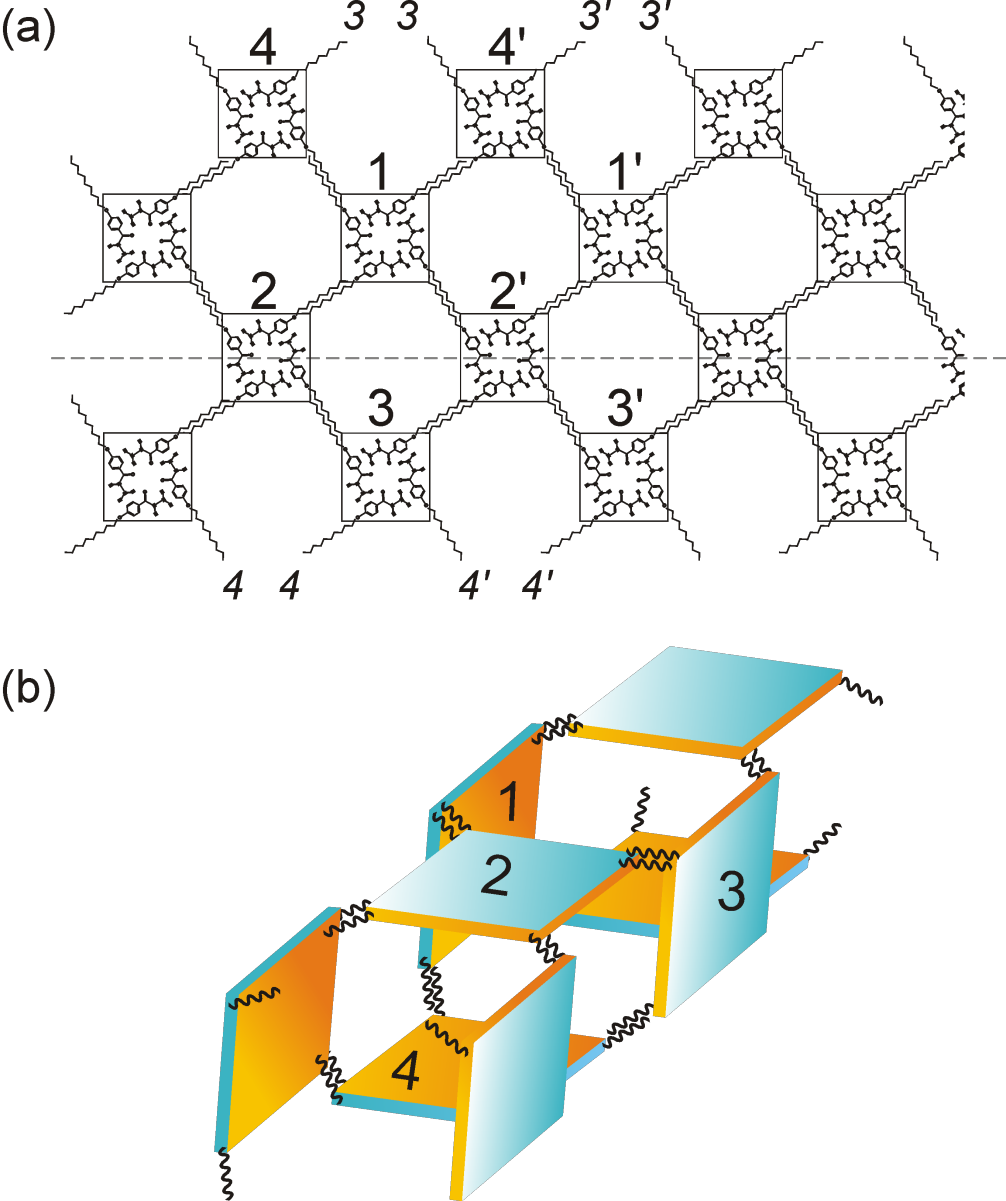
(a) Planar sheet (net) model (for representational purpose only) of a **1CHn-10** fibril section. The dashed line is drawn parallel to the fibril axis. (b) 3-D configuration for a three-strand fibril.

To visualise the structure of the **1CHn-10** fibril more clearly, a simplified model is shown in Figure 7. The “net” in Figure 7a (fibril dimensions are not drawn to scale for the 2-D representation) shows the interdigitation of alkyl chains between neighbouring tetramers in which the tetramer building blocks are represented by squares. The structure constituted by blocks 1, 2, 3 and 4 represents a repeating unit of the fibril. The aliphatic chains of the subsequent tetramers interact through van der Waals forces between the interdigitating chains. Thus, the capability of **1CHn-10** to achieve a fibril structure is based on its tendency to form tetramers, which is a crucial step in the process. The closure of the “net” is facilitated by the coupling between tetramers 3 and 3′ and tetramers 4 and 4′ and similarly all equivalent tetramers along the fibril, naturally defining the unique diameter of the fibril.

## Conclusion

We describe hitherto unexplored routes for the formation of mesophase-based, one-dimensional organic structures from hydrogen-bonded dimer/tetramer motifs. Linear structures are formed from planar molecular precursors by means of ring closure aided by van der Waals interactions between alkyl chains. The elementary fibrils of amphiphilic hydrazine derivatives observed at the liquid/solid interface show a distinctly different internal structure than the “disc-stacking” arrangement observed in the bulk. The molecular geometry is decisive in determining the precursors and eventually the structure of the elementary strands: dimer precursors for **2CHd-10** and tetramers for **1CHn-10**. While the internal dimer structure of **2CHd-10** fibrils allows fibrils of different diameters to be formed, **1CHn-10** fibrils are either simple linear chains of tetramers, or tetramers interweaved to form tubes with a fixed diameter. It follows that the large-scale morphologies at the liquid/solid interface are determined at the molecular/precursor level. Despite the compounds being specially designed as symmetric and asymmetric molecules, no inference is immediately discernible on the dependence of fibril structure on the symmetry, as it can easily be seen that the asymmetry of **1CHn-10** is broken at the precursor (tetramer) level. The precursor geometry rather than molecular symmetry determines the disparate fibril structures observed for the two investigated molecules.

## Supporting Information

A large-scale STM image of the area in Figure 6 and the height profile of the strands are available in the Supporting Information.

File 1 Large-scale STM image and height profile.
