# Supplementary material for: Dimer/tetramer motifs determine amphiphilic hydrazine fibril structures on graphite
Source: Beilstein J Nanotechnol. 2012 Sep 19;3:658–66. doi: 10.3762/bjnano.3.75 (PMC3458612; doi:10.3762/bjnano.3.75)
Supplement: File 1 — Large-scale STM image and height profile. [file Beilstein_J_Nanotechnol-03-658-s001.pdf]

Supporting Information

for

Dimer/tetramer motifs determine amphiphilic hydrazine fibril structures on graphite

Loji K. Thomas[1], Nadine Diek[2], Uwe Beginn[2] and Michael Reichling*[1]

Address: [1]Fachbereich Physik, Universität Osnabrück, Barbarastr. 7, 49076 Osnabrück, Germany and [2]Institut für Chemie, Universität Osnabrück, Barbarastr. 7, 49076 Osnabrück, Germany

Email: Michael Reichling* - reichling@uos.de

* Corresponding author

Large-scale STM image and height profile

Large-scale STM image and height profile of 1CHn-10 strands

The single-strand in Figure 6a of the main manuscript, with a rather low contrast, might be misperceived as a graphite step because single strands of **1CHn-10** are rarely observed compared to the three-strands. The image of the molecular fibril from Figure 6 has been taken in the area marked by the white arrow in Figure S1. The latter is a 350 × 350 nm^2 survey-scale image of the fibrils shown in Figure 6, taken at the solution–graphite interface. Figure S1b is another STM image of the same fibrils with a better contrast for the single strand. The height profile along the dashed black line, as well as the 3-D image (Figure S1c and Figure S1d, respectively), clearly illustrate the absence of any graphite step.

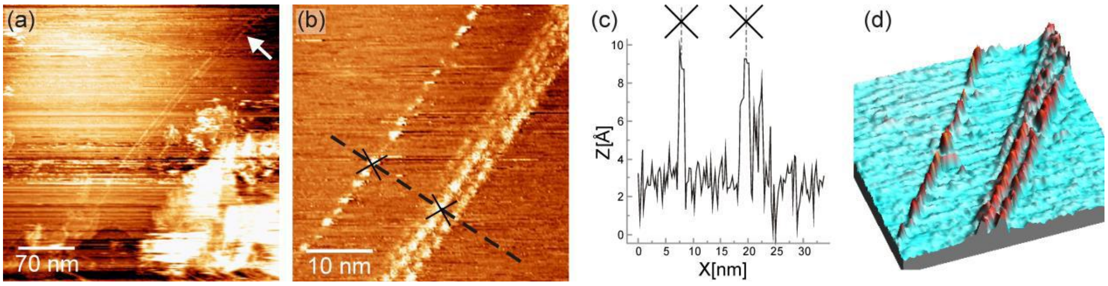

Figure S1: (a) Large-scale STM image showing the fibril at the liquid/solid interface. STM image of Figure 6a is taken near the region indicated by the white arrow. (b) Another STM image showing better contrast than Figure 6 for the single strand. (c) Height profile and (d) 3-D perspective of the STM image of (b) illustrating the absence of graphite steps.
